# Wide Complex Tachycardia in Patient With Cardiac Device

**DOI:** 10.1016/j.jaccas.2021.06.033

**Published:** 2021-09-01

**Authors:** Ana de Leon, Adrian Baranchuk, Andres Enriquez

**Affiliations:** Division of Cardiology, Queen’s University, Kingston, Ontario, Canada

**Keywords:** cardiac device, pacemaker, wide complex tachycardia, AV, atrioventricular, ECG, electrocardiogram, PMT, pacemaker-mediated tachycardia, PVAB, post-ventricular atrial blanking, PVARP, post-ventricular atrial refractory period, RVOT, right ventricular outflow tract, SVT, supraventricular tachycardia, WCT, wide complex tachycardia, VT, ventricular tachycardia

## Abstract

Electrocardiographic clues for a differential diagnosis of wide complex tachycardia in a patient with a pacemaker are presented. (**Level of Difficulty: Intermediate.**)

## Central Illustration

**Figure:**
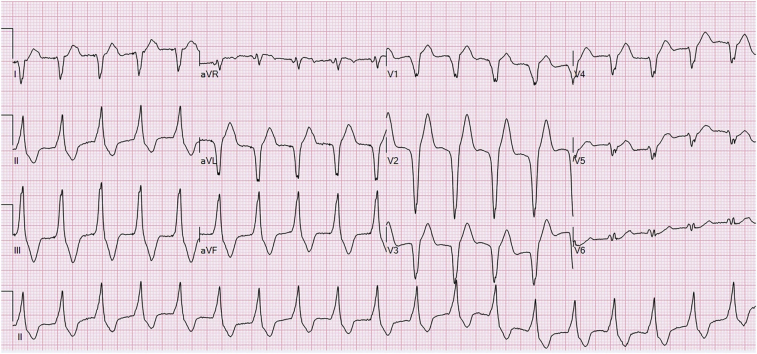

## CASE

**Figure 1:**
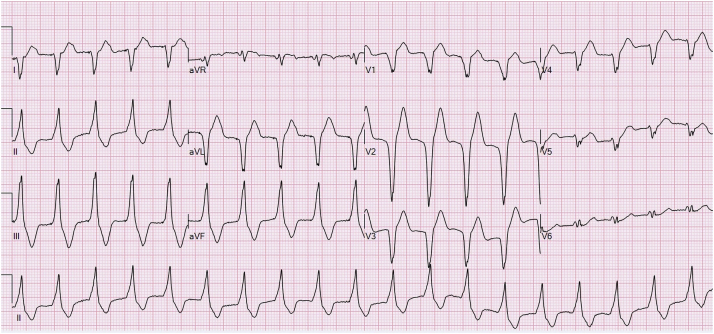
12-Lead Electrocardiogram of the Patient’s Tachycardia

An 85-year-old female patient was admitted to intensive care with a diagnosis of aspiration pneumonia. She had a medical background of hypertension, bioprosthetic aortic valve replacement for aortic stenosis, and implantation of a dual-chamber pacemaker for sick sinus syndrome. On admission, a sustained wide complex tachycardia (WCT) was observed on the monitor and documented on a 12-lead electrocardiogram (ECG) (Figure 1). The patient was asymptomatic and hemodynamically stable during the episode. Her device was in DDDR mode, had a lower/upper rate limit of 60/130 beats/min, a paced/sensed atrioventricular (AV) delay of 275/200 ms, a post-ventricular atrial refractory period (PVARP) of 275 ms, a post-ventricular atrial blanking (PVAB) of 150 ms, and a mode switch rate of 180 beats/min.

### WHAT IS THE DIAGNOSIS?

A. Supraventricular tachycardia (SVT) with left bundle branch block (LBBB)
B. Ventricular tracking of atrial tachycardia
C. Pacemaker-mediated tachycardia (PMT)
D. Ventricular tachycardia (VT)
E. Antidromic AV re-entrant tachycardia

The correct answer is C, but an acceptable answer is B.

## Explanation

The ECG shows a WCT at a rate of 115 beats/min. Main differential diagnoses should include VT, SVT with aberrancy, or pre-existing bundle branch block, tachycardia mediated by an accessory pathway, and ventricular paced rhythm. P waves are not clearly seen and may be hidden in the T-wave or QRS complex.

First, the QRS morphology (negative complex in leads I, aVL) is not typical for LBBB, excluding option A. The inferior QRS axis (positive in leads II, III, and aVF), with late precordial transition and negative deflections in leads aVR and aVL, indicates an origin from the right ventricular outflow tract (RVOT). No accessory pathway can produce such a pattern, and therefore, option E can also be ruled out. Careful examination of the ECG also reveals a small spike preceding every QRS complex, suggesting participation of the patient’s pacemaker in the wide QRS rhythm and making VT unlikely. Review of other available ECGs is helpful in these cases because they may show ventricular pacing that is identical to the tachycardia. The patient's chest x-ray in anteroposterior and lateral projections confirmed lead position in the RVOT, consistent with the paced morphology (Supplemental Figure 1A).

Once we determined that the wide QRS complex was pacing-mediated, the 2 possible options included a rapid atrial rhythm tracked by the device, or a PMT, in which retrogradely conducted P waves were tracked by the device, resulting in an endless loop tachycardia. A simple maneuver that can help to differentiate between both mechanisms is the application of a magnet over the device, which should interrupt a PMT or may help to understand the AV relationship by unmasking P waves in case of atrial tachycardia. This happens because the magnet triggers an asynchronous pacing mode, and the pacemaker will not sense atrial activity. In this case, pacemaker interrogation showed a normally functioning device. No atrial arrhythmias had been recorded and ventricular pacing through the programmer showed evidence of retrograde 1:1 ventriculo-atrial (VA) conduction, with a VA time of 309 ms (Supplemental Figure 1B). Therefore, PMT is the most likely mechanism of the tachycardia in this case. PMT always occurs at a rate equal or below the upper rate limit and its rate depends on the VA conduction time and the programmed sensed AV delay (1). Ventricular tracking of an atrial or sinus tachycardia cannot be ruled out with the available information. In support of option C, PMT was no longer observed after extending the PVARP. This is the interval after every ventricular event during which the atrial channel is refractory, preventing the inappropriate tracking of retrograde P waves (2).

## Funding Support and Author Disclosures

The authors have reported that they have no relationships relevant to the contents of this paper to disclose.
