# Supplementary material for: Wide Complex Tachycardia in Patient With Cardiac Device
Source: JACC Case Rep. 2021 Sep 1;3(11):1396–7. doi: 10.1016/j.jaccas.2021.06.033 (PMC8414536; doi:10.1016/j.jaccas.2021.06.033)
Supplement: Supplemental Figure 1 [file mmc1.docx]

Supplemental Figure 1

A. Chest X-ray AP and lateral projection.


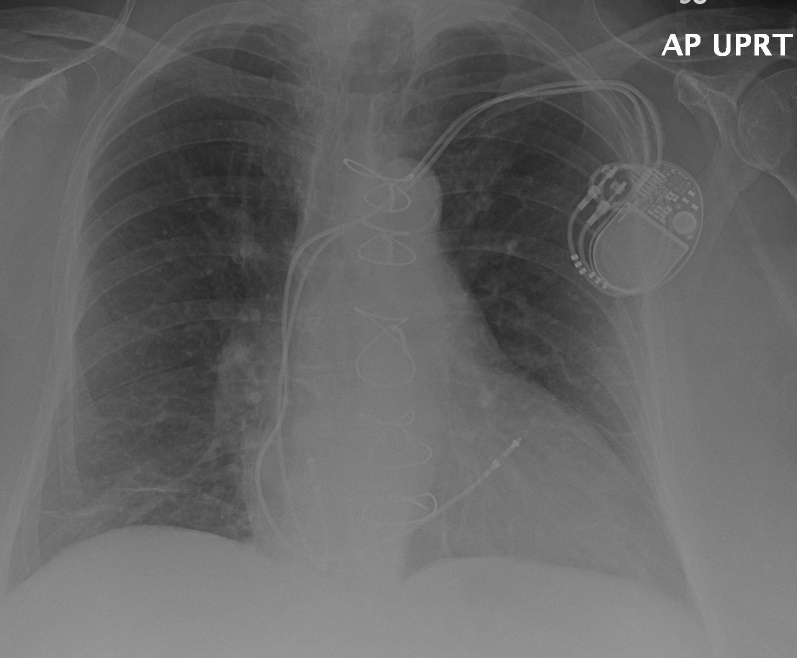

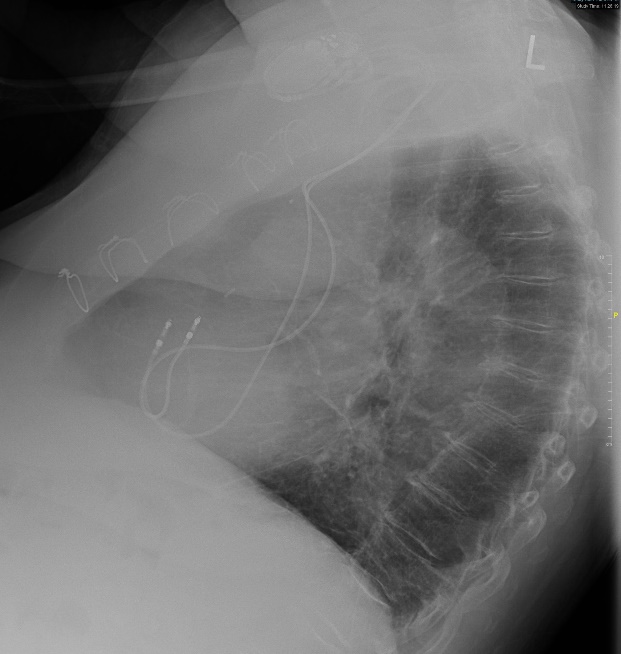


B. Intracardiac trace, measure of VA conduction time during right ventricular pacing
